# Novel approach to estimate respiratory quotient from the alveolar gas equation: a proof-of-concept study in pigs

**DOI:** 10.1186/s40635-026-00969-3

**Published:** 2026-09-08

**Authors:** Joakim Hedov, Josefin Elzén Junker, Magnus Hallbäck, Per-Arne Lönnqvist, Jacob Karlsson

**Affiliations:** 1 Department of Thoracic and Vascular Surgery, Linköping University Hospital, Garnisonsvägen 10, Linköping, 581 85 Sweden; 2 Department of Health, Medicine and Caring Sciences, Division of Diagnostics and Specialist Medicine, Linköping University, Linköping, 581 83 Sweden; 3 Thoracic Surgery Center, Karolinska University Hospital, Eugeniavägen 23, Stockholm, 171 64 Sweden; 4 Pediatric Perioperative Medicine and Intensive Care, Karolinska University Hospital, Eugeniavägen 23, Stockholm, 171 64 Sweden; 5 Department of Physiology and Pharmacology (FYFA), Lönnqvist Group, Section of Anesthesiology and Intensive Care, Karolinska Institute, Stockholm, C3, 171 76 PA Sweden; 6 Maquet Critical Care AB, Röntgenvägen 2, Solna, 171 06 Sweden

## Abstract

**Background:**

The respiratory quotient (RQ) reflects metabolic substrate utilization and is traditionally measured using indirect calorimetry techniques, e.g. Douglas bag, which are technically demanding and not always feasible in clinical practice. The alveolar gas equation (AGE) provides a theoretical relationship between oxygen tension, carbon dioxide tension and RQ and may be rearranged to estimate RQ using standard respiratory measurements. This study aimed to validate a novel method for estimating RQ by reversing the alveolar gas equation.

**Method:**

In this prospective experimental study, twelve anesthetized, mechanically ventilated pigs were studied under steady-state conditions. RQ was determined using two methods: (1) reference measurement via Douglas bag collection using Haldane transformation (RQref), and (2) calculation from the rearranged alveolar gas equation using inspired and expired gas measurements (RQcalc). Agreement between methods was assessed using paired t-tests, Bland-Altman analysis with bootstrap confidence intervals, and linear regression.

**Results:**

Mean RQref was 0.877 (95% CI 0.845–0.908), while mean RQcalc was 0.994 (95% CI 0.961–1.026), demonstrating a significant difference (*p* < 0.001). Bland-Altman analysis showed a bias of 0.117 (95% CI 0.103–0.131) with limits of agreement from 0.066 to 0.167 and a mean percentage error of 5%. A strong correlation was observed between methods (*r* = 0.895, *p* < 0.001), with RQcalc explaining 80% of the variance in RQref (R² = 0.801).

**Conclusion:**

Reversal of the alveolar gas equation enables estimation of RQ with strong correlation to reference measurements but introduces a consistent positive bias. While not directly interchangeable with Douglas bag measurements, this simplified approach may provide a practical, continuous approximation of RQ in settings where conventional metabolic monitoring is not feasible. Additional validation studies are needed to make this method ready for clinical use.

## Introduction

The respiratory quotient (RQ), defined as the ratio between carbon dioxide production (V̇CO_2_) and oxygen consumption (V̇O_2_), is a fundamental physiological variable that reflects the metabolic fuel being used by tissues at the cellular level [1]. In experimental and clinical physiology, RQ provides insight into metabolic substrate use, ranging from low values associated with mainly lipid oxidation (RQ ≈ 0.7) to high levels indicating carbohydrate oxidation (RQ ≈ 1.0). In addition, RQ serves as a key variable for estimating resting energy expenditure and oxygen delivery-consumption relationships [2]. Under controlled mechanical ventilation, changes in RQ may also signal alterations in ventilation-perfusion matching, shunt, or metabolic stress [3].

Traditionally, RQ is determined by indirect calorimetry or Douglas bag analysis of exhaled gases [4]. These methods, while accurate, are technically cumbersome, limiting routine use for intraoperative/intensive care or experimental applications [5].

The alveolar gas equation (AGE) initially described by Fenn et al. in 1946^6^ provides a link between alveolar oxygen tension (PAO_2_), inspired oxygen fraction (FiO_2_), alveolar carbon dioxide tension (PACO_2_), and RQ. The equation also includes the atmospheric pressure (P_atm_) and the water vapor partial pressure (P_H2O_) and is shown in a commonly used, but simplified form below (Eq. 1). Please note that Fenn et al. used the alveolar partial pressure of CO_2_ and not the partial pressure of CO_2_ in arterial blood, as is common in clinical practice today.

1 $$\:PA{O}_{2}=Fi{O}_{2}({P}_{atm}-{P}_{{H}_{2}O})-\frac{PAC{O}_{2}}{RQ}$$

This relationship forms the basis for estimating the alveolar-arterial oxygen gradient and assessing gas exchange abnormalities. However, under stable conditions, the equation can also be conceptually reversed: if FiO_2_, PAO_2_, and PACO_2_ are known, RQ becomes the only unknown variable. Solving the equation for RQ therefore allows a reversed estimation of the respiratory quotient.

Theoretically, such an approach would provide a real-time, non-invasive estimation of RQ using routinely available clinical measurements. However, this reversed alveolar gas equation calculation, has not been experimentally validated against established reference techniques.

We therefore developed and tested a novel method for estimating RQ based on the reversed alveolar gas equation. In this proof-of-concept study, we compared the estimated RQ values derived from inspired and expired gas measurements to reference values obtained using the Douglas bag method analyzed by a calibrated Datex gas analyzer (Datex‑Ohmeda Capnomac Ultima, GE HealthCare, Chicago, IL, USA). The experiments were conducted in a porcine model under mechanical ventilation with stable metabolic conditions to minimize confounding from metabolic variability and transient wash-in or washout of CO_2_ and O_2_ from the lungs and other body stores.

## Materials and methods

This study was conducted in 2024–2025 at the Hedenstierna laboratory in Uppsala, Sweden. A total of twelve domestic-breed pigs with median weight 28.8 kg (range 26.3–30.9), age 6–8 weeks and of mixed sexes, from a single breeding facility were included. The animals were housed under controlled light and temperature conditions, with free access to food and water. All experimental procedures were performed during daytime and adhered to the guidelines established by the Uppsala Animal Ethics Committee and the Animal Research: Reporting of In Vivo Experiments (ARRIVE) [7]. Ethics approval was obtained from Uppsala Animal Ethics Committee (reference 5.8.18–12744/2021, Jordbruksverket). The animals were also included in another experiment, estimating cardiac output with a capnodynamic method, in the presence of an aortopulmonary shunt [8], which was conducted after the metabolic measurements. The experimental setup followed the standard operating procedure for the laboratory, which has been described previously in greater detail [9].

### Anesthesia and monitoring

Induction of anesthesia was achieved by intramuscular injection of atropine (0.04 mg·kg^− 1^), tiletamine-zolazepam (6 mg·kg^− 1^), and xylazine (2.2 mg·kg^− 1^). Maintenance was intravenous and consisted of fentanyl (5 µg·kg^− 1^ bolus, followed by infusion 4 µg·kg^− 1^·h^− 1^), ketamine (30 mg·kg^− 1^·h^− 1^), midazolam (0.1 mg·kg^− 1^·h^− 1^), and rocuronium (1 mg·kg^− 1^ bolus, followed by infusion 2 mg·kg^− 1^·h^− 1^). The airway was established through a tracheostomy using a conventional cuffed endotracheal tube. Mechanical ventilation was administered in a volume-controlled mode with a Servo-i ventilator (Maquet, Solna, Sweden), set to deliver a tidal volume of 10 ml·kg^− 1^·and a fraction of inspired oxygen (FiO_2_) of 0.3. A positive end-expiratory pressure (PEEP) of 5 cm H_2_O was applied following a preliminary 2-minute lung recruitment maneuver performed at PEEP 10 cm H_2_O, maintaining constant driving pressure as previously described [9]. After recruitment, an air trial was conducted using FiO_2_ 0.21, and the recruitment procedure was repeated if necessary to sustain pulse oximetry values above 97%, confirming adequate pulmonary aeration [10].

Following anesthesia induction, a bolus of Ringer’s acetate (20 ml·kg^− 1^) was administered, after which maintenance infusions of glucose 25 mg·ml^− 1^ (8 ml·kg^− 1^·h^− 1^) and Ringer’s acetate (10 ml·kg^− 1^·h^− 1^) were started. Respiratory gas from the Y-piece was continuously sampled and analyzed by a connected gas monitor (Datex‑Ohmeda Capnomac Ultima, GE HealthCare, Chicago, IL, USA).

Additional monitoring consisted of electrocardiography, peripheral oxygen saturation with plethysmography, invasive blood pressure monitoring and an indwelling urinary catheter for monitoring of diuresis. Additionally, a central venous catheter was inserted for monitoring of central venous pressure and administration of drugs and fluids.

All animals were given a bolus dose of Heparin (5000 U; LEO Pharma) to reduce the risk of thromboembolic events associated with intravascular instrumentation.

The level of anesthesia and analgesia were regularly assessed throughout the experiment according to established laboratory protocols. Continuous assessment of sympathetic responses was performed by monitoring hemodynamic variations (i.e., changes in heart rate and blood pressure), reactions to eyelash stimulation, and motor responses to pain stimuli.

At the end of this and the following [8] experiment the animals were euthanized, while under general anesthesia, using intravenous potassium chloride (KCl: 100 mmol) via the central venous catheter as per established standard procedure in the laboratory [9].

### RQ measurement by douglas bag

Douglas bag collection and analysis of the expired gas mixture was used to calculate the respiratory exchange ratio (RER) which, in steady state, should equal the respiratory quotient (RQ). Measurements were obtained under stable experimental conditions and unchanged ventilator settings. Hemodynamic variables (heart rate, mean arterial pressure, central venous pressure) were monitored continuously and confirmed to be stable before sampling. Furthermore, we monitored the expired O_2_ and CO_2_ fractions using the side-stream analyzer and observed steady state conditions for the expired peak values. In addition, the continuous glucose-containing infusion was maintained at a constant rate throughout the experimental protocol. Both RER and RQ are expressed as the carbon dioxide elimination (V̇CO_2_) divided by the oxygen uptake (V̇O_2_) as described in Eq. 2.

2 $$\:RQ=RER=\frac{{\dot{V}CO}_{2}}{{\dot{V}O}_{2}}$$

V̇CO_2_ and V̇O_2_ were determined from the inspired oxygen fraction and mixed-expired gas samples using the Haldane transformation. The Haldane transformation uses the inertness of nitrogen (N_2_) and algebraic simplification to eliminate the need to measure the total volume of inspired and expired air, which would introduce measurement errors too large to make the method useful [11]. Assuming the inspired gas is free from CO_2_; V̇CO_2_ and V̇O_2_ can be expressed as fractions of inspired and expired volumes as shown in Eqs. 3 and 4, where Ve denotes expired volume, Vi inspired volume and Femix means fraction of the respective gas in the expired gas mixture.

3 $$\:{\dot{V}CO}_{2}=Ve\cdot\:{FemixCO}_{2}$$

4 $$\:{\dot{V}O}_{2}=Vi\cdot\:{FiO}_{2}-Ve\cdot\:{FemixO}_{2}$$

The amount of inspired and expired nitrogen must be the same, since nitrogen is an inert gas with zero net exchange. This is described mathematically in Eq. 5.

5 $$\:Vi\cdot\:{FiN}_{2}=Ve\cdot\:{FemixN}_{2}$$

Dividing by FiN_2_, the relationship between Vi and Ve is established (Eq. 6).

6 $$\:Vi=Ve\cdot\:\frac{{FemixN}_{2}}{{FiN}_{2}}$$

The nitrogen fractions can be expressed as:

7 $$\:Fi{N}_{2}=1-Fi{O}_{2}$$

8 $$\:Femix{N}_{2}=1-Femix{O}_{2}-FemixC{O}_{2}$$

Substituting the numerator in Eq. 2 with Eq. 3 and the denominator with Eq. 4 and then substituting Vi with Eq. 6 and replacing FiN_2_ and FemixN_2_ with Eqs. 7 and 8 yields Eq. 9.

9 $$\begin{aligned}\:RQ&=\frac{\dot{V}C{O}_{2}}{\dot{V}{O}_{2}}\\&=\frac{Ve\cdot\:FemixC{O}_{2}}{Ve\left(\frac{1-Femix{O}_{2}-FemixC{O}_{2}}{1-Fi{O}_{2}}\right)Fi{O}_{2}-Ve\cdot\:Femix{O}_{2}}\end{aligned}$$

Since Ve appears in both the numerator and denominator it can be cancelled. Further algebraic simplification gives the final expression (Eq. 10).

10 $$\:RQ=\frac{\left(1-Fi{O}_{2}\right)FemixC{O}_{2}}{\left(1-FemixC{O}_{2}\right)Fi{O}_{2}-Femix{O}_{2}}$$

Exhaled water vapor is not included in the above equations. Since water vapor increases the volume of the expired gas mixture and therefore reduces the fractions of the other gas components, it must be accounted for. The water vapor was therefore eliminated by drying the gas mixture before it entered the analysis chamber of the gas analyzer system. If the gas concentrations are reported in a dry state (ATPD, Ambient temperature and pressure, dry), then Eq. 9 can be used to determine the respiratory quotient (RQ) [11].

The procedure for each RQ measurement was as follows:

- To avoid dilution of the gas in the bag with inspiration gas, the ventilator bias flow during the expiration phase was turned off.
- FiO_2_ was decreased from 0.30 to 0.21. This was done to optimize the conditions for RQ calculation (as shown in Eq. 1), as well as ensuring a well-defined inspired gas concentration without depending on accurate gas mixing by the ventilator.
- To establish a steady-state of nitrogen (N_2_), a 20-minute wash-out of O_2_ was conducted after the change of FiO_2_ and prior to connecting the bag to the ventilator exhaust port.
- Gas was collected for approximately 10 min, during which roughly 70 L of expiratory gas were collected.
- The content of the bag was analyzed using a calibrated Datex gas analyzer (Datex-Ohmeda Capnomac Ultima, GE HealthCare, Chicago, IL, USA). Gas was sampled alternately from the bag and room air by manual gas switching at a rate of approximately 5 cycles per minute.
- Gas concentrations of FiO_2_ (room air), FemixO_2_ (bag) and FemixCO_2_ (bag) were measured at ATPD state (Ambient temperature and pressure, dry).

### Reversed alveolar gas equation (AGE)

RQ was estimated from inspired gas composition by rearranging the classical alveolar gas Eq. 1^2^ (Eq. 11) where PACO_2_ and PAO_2_ denotes the partial pressures of carbon dioxide and oxygen respectively in alveolar gas, FiO_2_ is the inspired fraction of oxygen, P_atm_ is the atmospheric pressure and P_H2O_ is the partial pressure of water vapor):

11 $$\begin{aligned}\:PA{O}_{2}=Fi{O}_{2}({P}_{atm}-{P}_{{H}_{2}O})-\frac{PAC{O}_{2}(1-{FiO}_{2}\left(1-RQ\right))}{RQ}\end{aligned}$$

By using dry gases P_H2O_ equals zero and can be removed from the equation. FiO_2_ multiplied by P_atm_ equals PiO_2_. Solving for RQ gives Eq. 12:

12 $$\:R\mathrm{Q}=\frac{PAC{O}_{2}(1-{FiO}_{2})}{Pi{O}_{2}-PA{O}_{2}-{FiO}_{2}\cdot\:{PACO}_{2}}$$

This expression (Eq. 12) is equivalent to the expression for RQ derived from the Douglas bag and the Haldane transformation in Eq. 10. The only difference is that alveolar gas is used in Eq. 12 and Douglas bag gas mixture (“emix”) in Eq. 10.

The procedure for the AGE-based RQ measurement was the same as for the RQ measurement by Douglas bag listed above, with the exception that gas sampling was done at the same rate as the ventilators respiratory rate, instead of 5 times per minute. Any measurement of FiCO_2_ > 0 was subtracted from PACO_2_ to compensate for gas analyzer lag at a higher respiratory rate. Simultaneous measurements for both methods of RQ estimation were conducted for each subject and used for comparison. The same gas analyzer (Datex-Ohmeda Capnomac Ultima, GE HealthCare, Chicago, IL, USA) was used for both methods.

### Statistical analysis

Raw data for absolute values of RQ for both methods as well as the differences between the methods, were controlled for normal distribution by the Shapiro-Wilk test and visual inspection of the corresponding histograms. Values are presented as mean and 95% confidence interval (CI).

The difference between RQ calculated from the reversed alveolar gas equation (RQ_calc_) and the reference RQ (RQ_ref_) from the Douglas bag measurements, was analyzed using a paired t-test. A p-value < 0.05 was considered statistically significant.

Agreement between RQ_calc_ and RQ_ref_ was assessed using Bland-Altman analysis, calculating bias (mean difference) and limits of agreement (± 1.96 SD). To increase statistical robustness, 95% confidence intervals for the bias and limits of agreement (LoA) were obtained using a nonparametric bootstrap procedure (10,000 resamples). For each bootstrap sample, bias and LoA were recalculated, and the 2.5th and 97.5th percentiles of the resulting distributions were taken as the 95% confidence intervals.

Pearson correlation and linear regression was used to quantify association between the methods.

Mean percentage error was defined as 1,96 × SD/mean of reference method.

A sample size of twelve animals, each with repeated steady-state observations, was considered adequate for method comparison in this proof-of-concept study.

All data were analyzed using R (version 4.5.0, R Core Team, Vienna, Austria) and Excel for Mac (version 16.107.4, Microsoft Corporation, Redmond, WA, USA) with the Real Statistics Resource Pack (Release 9.8, Copyright 2013–2026, Charles Zaiontz, www.real-statistics.com). Data are expressed as mean ± SD or median [IQR], as appropriate.

## Results

None of the animals showed signs of discomfort during the experiment, as assessed by stimuli described in the methods section.

RQ data for each subject in the cohort are displayed in Table 1 together with reference values for awake starved (24 h) and awake non-starved pigs [13, 14].

Averages were 0.877 (95%CI 0.845–0.908) for RQ_ref_ and 0.994 (95%CI 0.961–1.026) for RQ_calc_. There was a statistically significant difference between the RQ values obtained by Douglas bag and through the alveolar gas equation (*p* < 0.001).

**Fig. 1:**
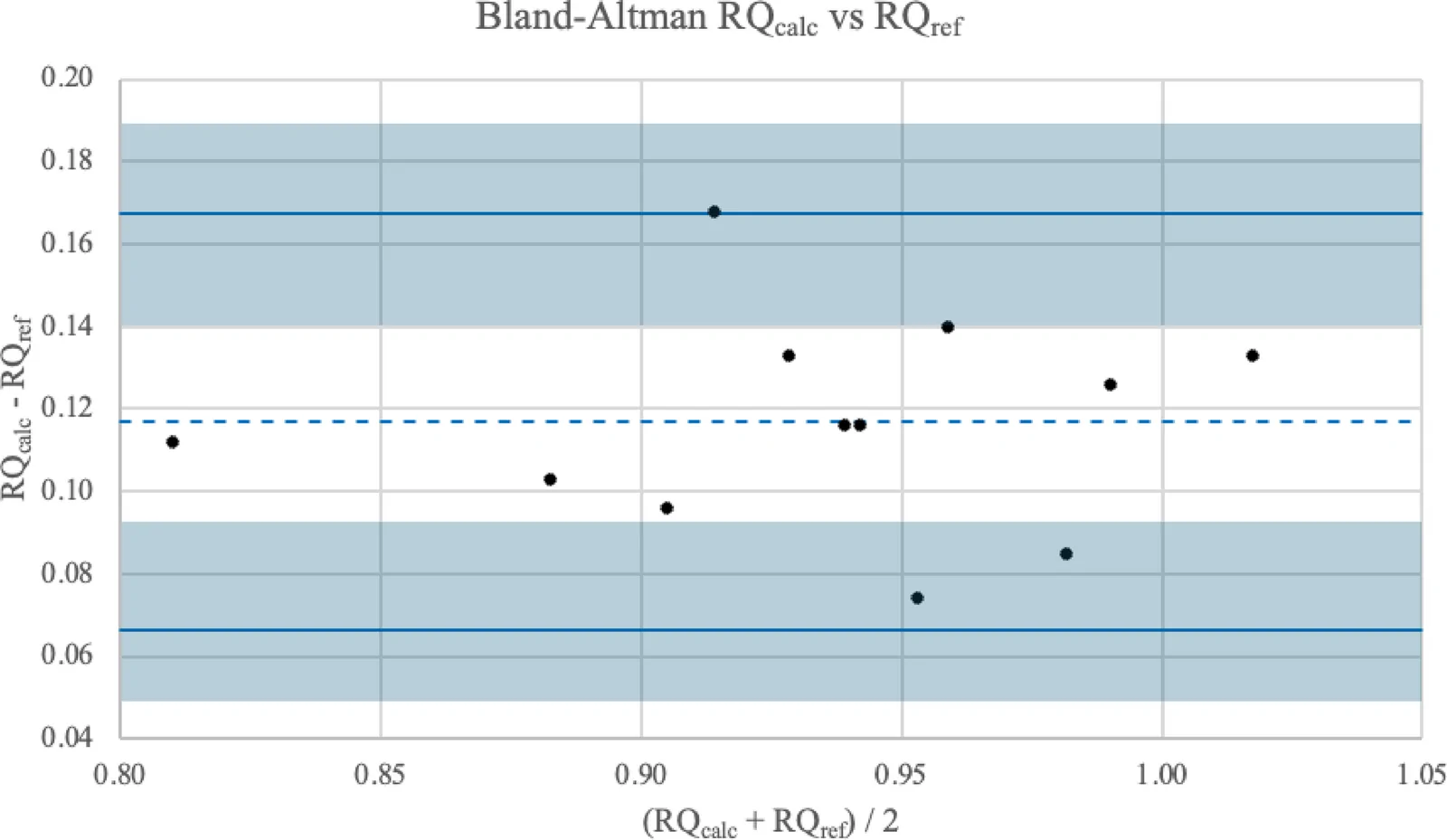
Bland-Altman plot displaying the agreement between the two methods. The X-axis plots the mean of the two methods for every paired measurement. The Y-axis plots the difference between the two methods for every paired measurement. Bias, expressed as the overall mean difference is illustrated by the blue broken line. Upper and lower levels of agreement are shown as blue solid lines. The blue shaded areas represent the 95% CIs of the upper and lower levels of agreement. N=12

Bland-Altman analysis, plotted in Fig. 1, showed a bias of 0.117 (95%CI 0.103–0.131) with 95% limits of agreement 0.066 (95%CI 0.049–0.093) to 0.167 (95%CI 0.140–0.189) and a mean percentage error of 5%.

**Fig. 2:**
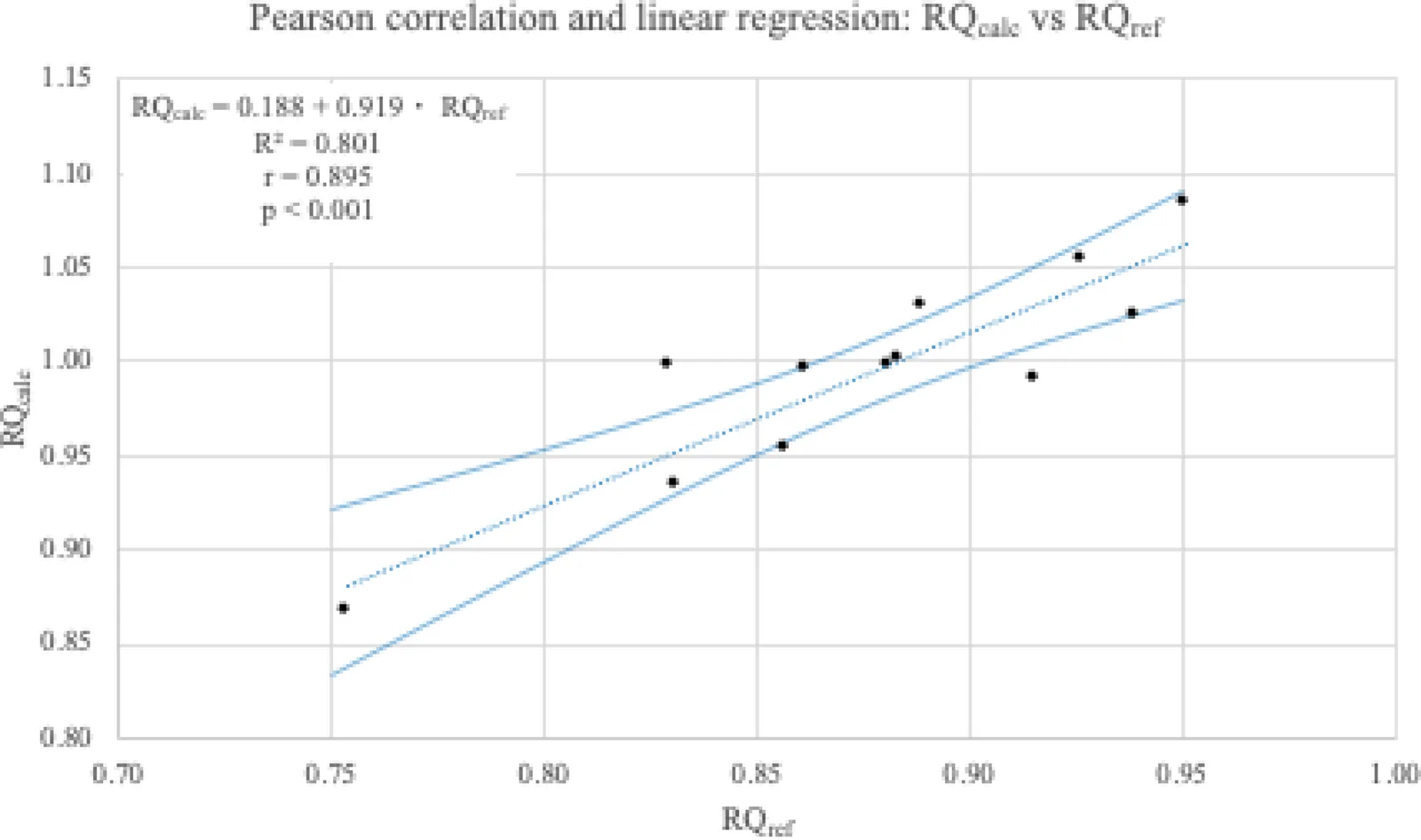
Correlation and linear regression between the two methods. The dotted line represents the fitted linear regression line and the solid lines the 95% confidence interval. Pearson’s correlation coefficient was r = 0.895 (p < 0.001), with R2 = 0.801

Pearson’s correlation showed a positive relationship between RQ_ref_ and RQ_calc_ (*r* = 0.895, *p* < 0.001). Simple linear regression indicated that RQ_calc_ increased by 0.919 for each 1.000 increase in RQ_ref_ with the intercept at 0.188. The regression model explained 80.1% of the variance in RQ_calc_ (R² = 0.801). Both Pearson’s correlation and linear regression are displayed in Fig. 2.

**Table 1:** Individual RQ values obtained with both reference method (Douglas bag) and tested method (Alveolar gas equation)

	RQ_ref_ (Douglas bag)	RQ_calc_ (Alveolar gas equation)
Pig01	0.831	0.934
Pig02	0.884	1.000
Pig03	0.881	0.997
Pig04	0.916	0.990
Pig05	0.927	1.053
Pig06	0.889	1.029
Pig07	0.951	1.084
Pig08	0.754	0.866
Pig09	0.862	0.995
Pig10	0.830	0.998
Pig11	0.939	1.024
Pig12	0.857	0.953
Mean (95%CI)	0.877 (0.845–0.908)	0.994 (0.961–1.026)
RQ awake pig starved 24 h	0.87	
RQ awake pig non-starved	1.07	

## Discussion

In this proof-of-concept study, we evaluated whether the respiratory quotient could be estimated by reversing the alveolar gas equation and compared with reference measurements obtained via Douglas bag analysis. The reversed equation produced RQ values that correlated strongly with the reference method (*r* = 0.895) but consistently overestimated RQ. This was also demonstrated in the Bland-Altman plot, showing a positive bias of 0.117, but with narrow limits of agreement and a low mean percentage error. Despite this systematic offset, the calculated RQ values remained physiologically plausible across the entire range observed, suggesting that the method captures true variability in metabolic state. These findings indicate that, while the reversed alveolar gas equation does not necessarily provide interchangeable values with Douglas bag measurements, it may still hold value as a simple and continuous approximation of RQ in settings where traditional metabolic monitoring is impractical.

### Limitations

A possible source of error in this setup is the probability of a different response time for CO_2_-analysis as compared to the O_2_-analysis within the gas analyzer. Assuming the O_2_-analyzer is slightly slower, the respiratory rate of 20 (for the AGE-derived values) may have overestimated the alveolar O_2_, resulting in a lower value in the denominator in Eq. 12, which yields a higher RQ, which would explain a systematic bias.

In the original article by Fenn et al.^6^, another version of the alveolar gas equation is presented for use when FiCO_2_ > 0. This equation was also tested in this experiment but was found to produce results of negligible difference (i.e. RQ difference approximately 0.0001).

In this study, RQ values were obtained under exceptionally stable and controlled conditions that may not be reproducible in typical clinical settings. This study did not examine the ability of the AGE-derived RQ to track changes in metabolic activity over time, such as those induced by prolonged starvation or by metabolic manipulation (i.e. insulin or epinephrine infusion).

The respiratory exchange ratio (RER) was considered equivalent to the respiratory quotient (RQ), which is reasonable under steady-state conditions. However, RQ strictly refers to substrate oxidation at the cellular level, which was not directly assessed. Instead, RQ was estimated from airway gas exchange, and therefore represents a gas-exchange derived respiratory exchange ratio rather than a direct measure of cellular metabolic activity. Equivalence between these measures can only be assumed under steady-state conditions of ventilation and metabolism and this must be taken into consideration.

Fractions of inspired and expired O_2_ and CO_2_ in this study, were acquired by extracting maximum and minimum values from the waveform from the gas analyzer. For the Douglas-derived RQ this was done five times per minute, but for the AGE-derived RQ this had to be done at the same rate as the tidal-ventilation of the pigs, which was 20 breaths per minute. It is possible that the gas analyzer was not fast enough to correctly read samples at this higher rate, which may have introduced a systemic error, illustrated as the systemic bias between the methods. A slower respiratory rate may potentially correct this even if this was not investigated in detail.

It is important to note that the Douglas bag technique, considered the reference method here, has its own limitations, primarily related to the need for highly precise gas analysis. This introduces uncertainty when interpreting individual RQ values, despite the suitability of both techniques for estimating RQ at a population level. Furthermore, precision assessments are difficult to achieve with Douglas bag or other gas-mixing chamber systems, meaning that their individual-level accuracy is inherently uncertain. Previous work has demonstrated considerable variability between different types of gas-mixing chambers, further highlighting these uncertainties [15]. Thus, while the Douglas bag served as the reference in this study, the accuracy of all gas-mixing chamber methods ultimately depends on the reliability of the underlying gas analysis and the ability to maintain a stable fraction of inspired oxygen.

The Datex Deltatrac metabolic monitor, often considered a reference method for indirect calorimetry, has been shown to differ from mass spectrometry with a bias of 0.018 and limits of agreement from approximately − 0.03 to 0.066 (i.e., a range of approximately 0.1) for respiratory quotient in mechanically ventilated subjects [16]. Thus, even this established technique showed an agreement range of nearly 0.1 against gold standard mass spectrometry. These findings support that disagreement between methods is inherent to respiratory quotient measurement rather than unique to the present approach.

Ideally, a truly independent reference method, such as mass spectrometry, would have been used as the reference method; however, this was not feasible due to practical constraints in the present study. On the other hand, since the same gas analyzer was used for both methods, it is easier to isolate the source of error, i.e. that the difference in RQ may arise from different sampling techniques, and potentially correct these errors in future research.

Yet another limitation is that we used only FiO_2_ = 0.21 (room air) in this study. There were mainly three reasons for doing so. First, the lower the FiO_2_, the less sensitive the RQ calculation is to gas measurement errors. Since the calculation is based on the presence of N_2_ (Haldane), more N_2_ gives better robustness. Second, the composition of the inspiratory gas is more accurate when only air is delivered. For higher FiO_2_, which may be used during ventilator therapy, the performance of the ventilator’s delivery of air and O_2_ mixture will influence the results. Third, when analyzing the content of the Douglas bag we could determine mixed expired gas and mixed inspired gas by alternating the gas sampling between bag gas and ambient air (done manually at a rate of about 5 times per minute).

### Potential clinical application

This study provides a proof-of-concept for a simplified approach to RQ estimation based on the alveolar gas equation. In the intensive care environment, RQ assessment is often used for metabolic monitoring and nutritional guidance and typically requires specialized metabolic monitors and trained personnel [17]. If RQ can be estimated with reasonable accuracy under stable conditions using gas sampling during tidal ventilation, this may represent a practical simplification.

## Conclusion

Reversal of the alveolar gas equation provides a simple method to estimate respiratory quotient under steady-state conditions. Although the method showed a strong association with the reference technique, the systematic positive bias may limit direct interchangeability with the applied reference method (Dougals bag). This approach may however offer a practical alternative for RQ estimation when conventional metabolic monitoring is not feasible. Additional validation studies are needed to make this method ready for clinical use.

## Data Availability

The data that support the findings of this study are available from the corresponding author upon reasonable request.
